# Hematologic Malignancies Diagnosed in the Context of the mRNA COVID-19 Vaccination Campaign: A Report of Two Cases

**DOI:** 10.3390/medicina58070874

**Published:** 2022-06-30

**Authors:** Maria-Alexandra Zamfir, Liliana Moraru, Camelia Dobrea, Andreea-Elena Scheau, Simona Iacob, Cosmin Moldovan, Cristian Scheau, Constantin Caruntu, Ana Caruntu

**Affiliations:** 1 Department of Oral and Maxillofacial Surgery, “Carol Davila” Central Military Emergency Hospital, 010825 Bucharest, Romania; ma.zamfir23@gmail.com (M.-A.Z.); liliana.moraru@yahoo.com (L.M.); ana.caruntu@gmail.com (A.C.); 2 Department of Oral and Maxillofacial Surgery, Faculty of Dental Medicine, “Titu Maiorescu” University, 031593 Bucharest, Romania; 3 Department of Hematology, Fundeni Clinical Institute, 022328 Bucharest, Romania; cameliadobrea@yahoo.com; 4 Department of Hematology, University of Medicine and Pharmacy “Carol Davila”, 050474 Bucharest, Romania; 5 Department of Radiology and Medical Imaging, Fundeni Clinical Institute, 022328 Bucharest, Romania; andreea.ghergus@gmail.com; 6 Pathology Laboratory Personal Genetics, 010987 Bucharest, Romania; simona.iacob@personalgenetics.ro; 7 Faculty of Medicine, “Titu Maiorescu” University, 031593 Bucharest, Romania; moldovan.cosmin@gmail.com; 8 General Surgery Ward, Witting Clinical Hospital, 010243 Bucharest, Romania; 9 Department of Physiology, “Carol Davila” University of Medicine and Pharmacy, 050474 Bucharest, Romania; 10 Department of Dermatology, “Prof. N.C. Paulescu” National Institute of Diabetes, Nutrition and Metabolic Diseases, 011233 Bucharest, Romania

**Keywords:** lymphoma, COVID-19, SARS-CoV-2 mRNA-based vaccine

## Abstract

Background: During the last two years, the COVID-19 pandemic led to millions of disease-related deaths worldwide. The efforts of the scientific community facing this global challenge resulted in outstanding achievements. Thus, within one year, new mRNA-based vaccines against SARS-CoV-2 viral infection were released, providing highly efficient protection and showing a very good safety profile in the general population. However, clinical data collection after vaccination is a continuous process for the long-term safety of any new medical product. The aim of our paper is to present two cases of hematological malignancies: diffuse large B-cell non-Hodgkin lymphoma and T/NK-cell lymphoma, diagnosed shortly after the administration of the mRNA COVID-19 vaccine. Methods and Results: Case 1: A female patient was admitted with a suspicious cervical mass that emerged within one week after the administration of second dose of the BNT162b2 COVID-19 vaccine. Surgical removal followed by pathology assessment of the specimen confirmed the diagnosis of diffuse large B-cell non-Hodgkin lymphoma. Case 2: A male patient was admitted with multiple ulcerative oral lesions arising on the third day after the initial dose of the BNT162b2 COVID-19 vaccine. These lesions had a progressive character and during the following months were complicated with repetitive episodes of heavy oral bleeding, requiring blood transfusions. The incisional biopsy of the lesions and pathological assessment of the specimens confirmed the diagnosis of T/NK-cell lymphoma. Conclusions: The safety profile of the mRNA-based vaccines is an undeniable fact. In most cases, suspicions of potentially aggressive side effects were ruled out, proving to be transient post-vaccine reactions. Clinicians should remain alert to report any potentially aggressive manifestations emerging in the context of mRNA COVID-19 vaccination, such as these cases of hematological malignancies, in order to promote additional investigations on the particular mechanisms of action of COVID-19 vaccines and to provide the best medical care to the patients.

## 1. Introduction

Since 2019, the novel coronavirus has infected more than 400 million people globally, with over 5 million associated deaths, changing the world as we knew it [1]. The resolution of such a crisis could be expected when herd immunity against this extremely contagious SARS-CoV-2 virus is achieved, either through vaccination or through recovering from the disease [2]. Taking these facts into consideration, the COVID-19 outbreak has presented members of the scientific community with a challenge: to design an efficient vaccine as soon as possible–and so they did. Within one year after the first reported case of COVID-19 [3], specific vaccines were developed. Between 11–18 December 2020, the US Food and Drug Administration (FDA) issued Emergency Use Authorisations (EUA) for two vaccines based on messenger RNA (mRNA) technology to protect against the COVID-19 disease: the BNT162b2 mRNA COVID-19 vaccine (Pfizer-BioNTech) and the mRNA-1273 COVID-19 vaccine (Moderna), with the recommendation for a two-dose regimen [4,5]. Shortly afterward, intense vaccination campaigns began in many countries worldwide, leading to the current status of a fully vaccinated population exceeding 4 billion people [1]. Initially, the vaccination program generated concerns regarding this novel technology based on genetic material, and encountered resistance in many countries worldwide [6]. One of the main concerns in the general population was the risk of potentially unknown side effects of the vaccine, especially long-term side effects, related to the rapid development and registration process [7]. However, the collection of safety and efficacy data is a continuous process for all pharmaceutical products and devices, including the novel mRNA COVID-19 vaccines, and nowadays, after more than a year since the release of the vaccine, the scientific community provides highly reliable data regarding the safety and efficacy of COVID-19 mRNA vaccines [8,9,10].

One of the major concerns regarding these vaccines was related to the link between mRNA vaccines and malignancy, especially hematologic malignancies. This is because these vaccines, similarly to other types of vaccines, stimulate immune cells in generating protection against a future contact with a specific pathogen. The currently available data provide strong support for the benefits of anti-COVID-19 vaccination in patients with solid cancers, including patients undergoing immunosuppressive therapies [11]. In these patients, increasing levels of neutralizing antibodies were detected with every additional dose of immunization and there were no significant side effects compared to healthy controls [12,13]. In patients with hematological cancers, efficacy rates were significantly lower after a two-dose regimen of mRNA vaccine administration. Active systemic treatment was associated with the highest risk for non-response. Nevertheless, the safety profile of the mRNA vaccine revealed no differences between the study groups, confirming the safety of mRNA vaccination in hematological patients [14]. Similar findings were reported in another study conducted on patients diagnosed with B-cell non-Hodgkin lymphoma [15]. After billions of vaccinations, only isolated cases of exacerbation or recurrence of pre-existing hematologic malignancies shortly after COVID-19 vaccination have been reported for both viral vector- and mRNA-based vaccines [16,17]. Even if in a worldwide vaccination campaign such isolated events can occur coincidentally, they must be reported, thus inviting additional investigations on the particular mechanisms of action of COVID-19 vaccines in this category of patients.

In this paper, we present two cases of hematologic malignancies: diffuse large B-cell lymphoma and T/NK-cell lymphoma, diagnosed shortly after the administration of the mRNA COVID-19 (Pfizer-BioNTech) vaccine.

## 2. Case Report

**Figure 1:**
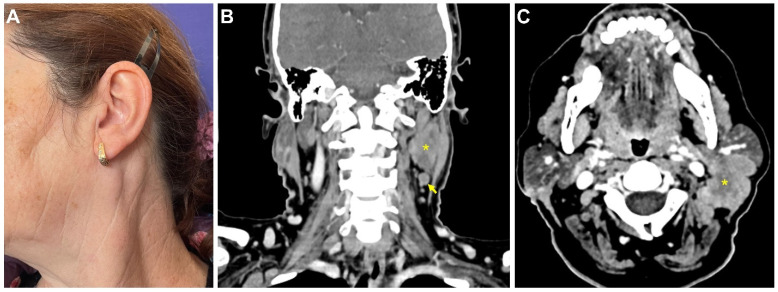
Clinical and imaging aspects at presentation. Clinical aspect (**A**). Contrast-enhanced CT scan of the neck in the coronal (**B**) and axial (**C**) planes. Large necrotic tumor (*) located on the posterior and inferior aspect of the left parotid gland, with infiltration of the adjacent muscles and enlarged regional lymph nodes (arrow).

**CASE 1:** In October 2021, a 58-year-old female patient was referred to the Department of Oral and Maxillofacial Surgery of the “Carol Davila” Central University Emergency Military Hospital, with complaints of a painful round mass in the upper left cervical area. Clinical examination revealed a tumor mass at the angle of the left parotid gland, firm and painful on palpation, fixed on the underlying tissue, with a diameter of approximately 4 cm (Figure 1A). The patient was recently vaccinated for protection against COVID-19 infection, with a two-regimen mRNA vaccine (Pfizer-BioNTech)—the first dose on 22 May 2021 and the second dose on 12 June 2021. The patient reported that one week after the second dose inoculation, she noticed the sudden development of this painful cervical mass, which was small at the beginning and was associated with a flu-like syndrome: sore throat, odynophagia, headache, and rhinorrhea. The patient took over-the-counter anti-inflammatory drugs, with complete resolution of the flu-like symptoms within a week, except for the cervical mass, which continued to grow slowly.

The medical history of the patient revealed two natural births, an appendectomy in childhood, and thrombophlebitis, treated with stripping of the superficial varicose veins at both lower limbs five years prior with permanent antithrombotic medication (250 Sulodexide Lipasemic Units (LSU) BID and bioflavonoid 500 mg BID). She was also receiving long-term treatment for hypercholesterolemia (simvastatinum, 20 mg QD). The patient was not diagnosed with any other chronic or severe disease and denied personal or family history of malignancy. She was an active person, employed, with no exposure to toxic substances. She denied smoking and alcohol consumption. During the annual medical check-up visits, no clinical or paraclinical abnormalities were identified, except for increased cholesterol levels. She also denied other symptoms, such as weight loss, recurrent fever, chronic fatigue, or loss of appetite.

Cervical ultrasonography, recorded one month before presentation (in September 2021), objectified a well-defined, heterogeneous hypo- and hyperechoic nodular mass, located in the upper left cervical region, measuring 35.5/20/26 mm. The lesion was in direct contact with the posterior contour of the parotid gland and had a weak Doppler signal in the periphery, suggestive of a benign tumor. Several reactive lymph nodes with a maximum diameter of 17.6/8.3 mm were found adjacent to the inferior contour of the lesion. The contrast CT scan revealed a tumor mass located at the posteroinferior parotid gland contour, with irregular margins, measuring 38/26/32 mm with posterior infiltration into the paravertebral muscles and necrotic areas within the lesion, suggestive of malignancy (Figure 1B,C). Multiple lymph nodes in levels IIA and IB with a maximum diameter of 8 mm were described. There were no other abnormal findings.

**Figure 2:**
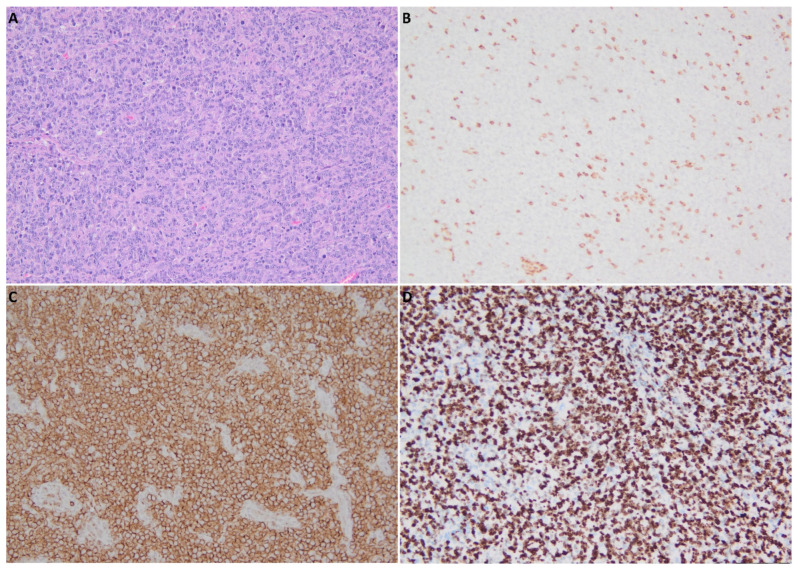
Histopathology and immunohistochemistry assessments: (**A**) Large size, lymphoid-type cells with increased rates of mitotic activity, HE, original magnification ×20; (**B**) positive staining for CD3 in reactive T cells, original magnification ×20; (**C**) intense and diffuse positive CD20 staining in tumor cells, original magnification ×20; (**D**) cellular proliferation index ki67, original magnification ×20.

In this context, considering the suspicion of malignancy, the indication was for exploratory and diagnostic surgery. Preoperative assessment included general blood tests, ECG, and cardiac and pulmonary evaluations, with no other findings besides the increased cholesterol level. The surgical procedure, conducted under general anesthesia with orotracheal intubation, started with an initial biopsy and frozen section examination of the specimen, which confirmed the presence of malignant proliferation within a lymph node. Radical tumor resection, including part of the paravertebral muscles, and left modified neck dissection, corresponding to levels I, II, and VA, were undertaken. Postoperatively, the patient received intravenous antibiotic therapy, and analgesic and anti-inflammatory drugs (amoxicillin and clavulanic acid 1 g, paracetamol 10 mg/mL, metamizole 5 mL BID) during the hospitalization period. The patient underwent the standard postoperative care protocol: daily wound cleaning with antiseptic solutions, removal of the draining tube after 24 h (drainage < 20 cc/24 h), and wound dressing. Postoperative recovery was uneventful, and the patient was discharged 48 h after surgery with an antibiotic prescription for the following three days (amoxicillin and clavulanic acid 1 g BID) and pain killers as needed. The sutures were removed after one week. At the three-week follow-up visit, wound healing was optimal. The pathology report outlined architectural changes within the left jugulodigastric lymph node (level IIA), necrosis and tumor infiltration with large size, lymphoid type cells showing increased rates of mitotic activity, suggestive of large-cell non-Hodgkin lymphoma (Figure 2A). Similar changes were found only in two lymph nodes corresponding to levels IIB and V, while the rest of the examined nodes exhibited changes suggestive of reactive histiocytosis. Immunohistochemistry (IHC) was recommended to confirm the diagnosis. IHC staining was intense and diffuse positive for CD20 and nuclear positive for PAX5 in tumor cells. The cellular proliferation index ki67 was between 80–85%. Positive staining for CD3 was seen in reactive T cells dispersed between tumor cells. IHC staining was negative for CD30, AE1/AE3 (Figure 2B–D). Both microscopy and IHC aspect were compatible with a malignant B-cell proliferation, specifically diffuse large B-cell non-Hodgkin lymphoma (DLBCL-WHO/IARC ICD-O: 9680/3).

After the surgery, the patient was referred to the hematology department for further investigation and treatment. An osteomedullar biopsy was conducted for disease staging. Both hematoxylin-eosin (HE) staining and IHC revealed medullary tissue with age-related specific changes and minimal reactive lymphocytosis. The PET-CT scan, performed one month after the surgery, did not reveal any metabolic activity suggestive of malignancy. All general clinical assessments and laboratory tests were within normal parameters. In this case, the treatment consisted of local radiotherapy: 20 sessions administered 5 days per week and a follow-up program, which is currently ongoing. The oncology board informed the patient of the standard of care represented by combined treatment: chemotherapy plus radiotherapy and alternative options of treatment [18], as well as the potential consequences, but she refused chemotherapy.

**Figure 3:**
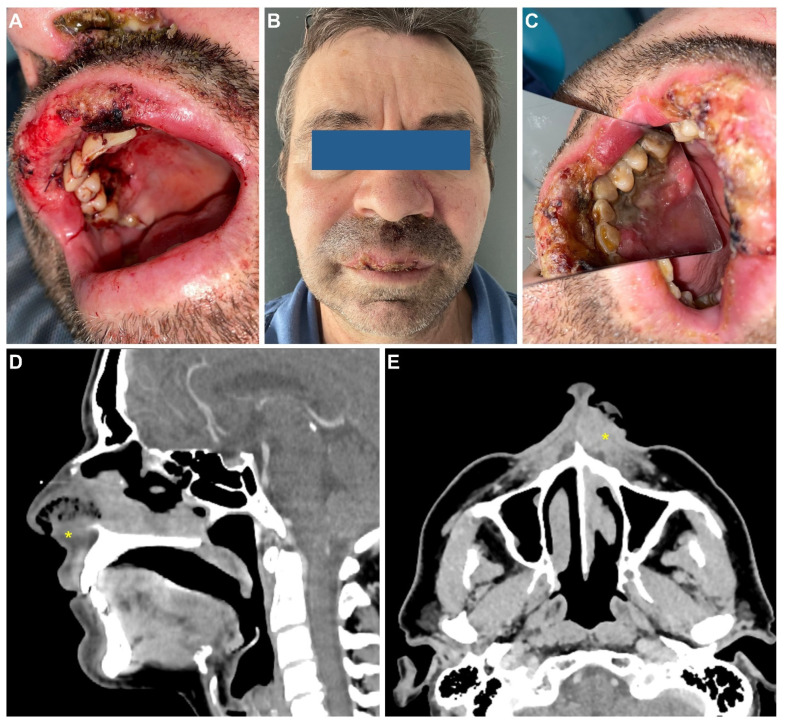
Clinical and imaging aspects at presentation. Intraoral aspect at the presentation to the emergency room, after superior labial artery ligature (**A**); clinical appearance after 2 days (**B**,**C**); contrast-enhanced CT scan of the neck in the sagittal (**D**) and axial (**E**) planes. Heterogeneous infiltrative mass (*) of the upper lip, left inferior nasal vestibule, and left inferior turbinate.

**CASE 2**: In December 2021, a 53-year-old male patient was referred to the Department of Oral and Maxillofacial Surgery of the “Carol Davila” Central University Emergency Military Hospital. Clinical examination revealed several erosive, overlapping, and painful lesions located on the mucosal surface of the upper lip, with diameters up to 5 mm, and normal surrounding mucosa. The lesions were progressing for about one month. In addition, the clinical assessment revealed very poor oral hygiene. The patient had recently completed a two-dose vaccination regimen against COVID-19 with the mRNA BNT162b2 vaccine: the first dose on 6 November 2021 and the second dose on 28 November 2021. Reported post-vaccination reactions were fever up to 39.5 degrees and shivering episodes during the first 24 h. On the third day after the first dose, the patient noticed the appearance of a painless ulcer on the mucosal surface of the upper lip. He initiated self-treatment with topical vitamin A. The lesion extended and new lesions developed, in spite of the therapy. Meanwhile, the second dose of the vaccine was administered and, because the oral lesions were progressing, the patient sought specialized medical assistance. The medical history revealed that the patient was under treatment for hypertension for several years (metoprolol tartrate 50 mg TID) and for anxiety (Venlafaxine 25 mg BID). He was not diagnosed with any other chronic condition and denied a personal or family history of malignancy. The patient was a construction worker and reported long-term exposure to industrial dust. He also had a 25-year history of heavy smoking (20 cigarettes/day), with complete cessation about 8 years prior. The patient denied alcohol or drug abuse. He did not complain of other symptoms, such as weight loss, fatigue, or loss of appetite, at the first visit to our department. The clinical suspicion was of infection (with herpes simplex virus) or a bullous disease with oral manifestations. The patient was referred to dermatology for further investigations and treatment. One week later, the patient was brought to the emergency room with active oral bleeding. Clinical examination revealed extensive oral ulcers involving the upper lip vermilion and mucosa, right side gingiva, and palatal mucosa, with bleeding areas and necrotic areas, intense pain, as well as swelling of the upper lip. Furthermore, new areas of necrosis covered with organic debris were identified in the left nostril (Figure 3A–C). Blood tests revealed severe anemia (hemoglobin 7.0 g/dL) with iron deficit (46 µg/dL), elevated inflammatory markers (fibrinogen- 549 mg/dL), ESR (23 mm/1 h) and increased platelet numbers (478 k/µL).

**Figure 4:**
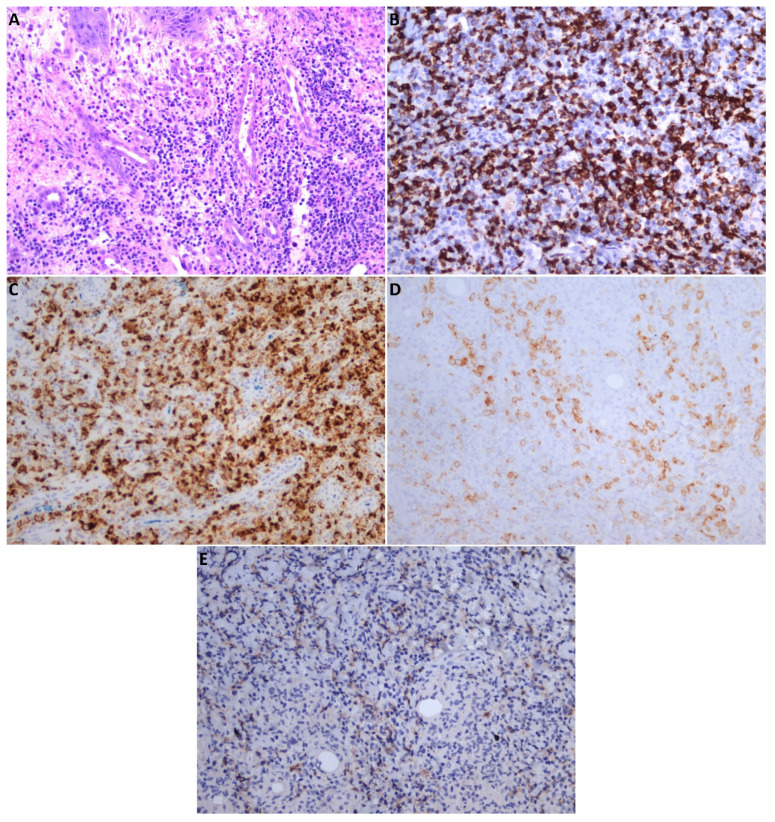
Histopathology and immunohistochemistry assessments: (**A**) polymorphous lymphoid infiltrate in the epithelium, with small- or medium-sized cells, with incised or angulated nuclei and moderate cytoplasm, HE, original magnification ×20; (**B**) diffuse positive staining for CD3 in T cells, original magnification ×20; (**C**) diffuse positive staining for granzyme B (a marker for cytotoxic activation), original magnification ×20; (**D**) positive staining for CD30 (a marker for activated lymphocytes), original magnification ×20; (**E**) cells with low-intensity staining for CD56 (a marker for NK cells), original magnification ×20.

The patient was admitted to our department for emergency treatment. Under local anesthesia, the source of active bleeding was identified as the superior labial artery and was ligatured; the patient received a transfusion with one unit of blood. Diagnostic investigations were initiated. The patient tested negative for COVID-19, hepatitis B and C, and HIV. A bacterial swab from the ulcer revealed infection with *Pseudomonas aeruginosa*, sensitive to all types of antibiotics. Immunology tests revealed abnormally elevated values for C4 serum complement and serum Ig E (43.9 mg/dL and 536.6 UI/mL, respectively). No other abnormal findings were detected after the general assessment. The head and neck contrast CT scan revealed reactive bilateral cervical lymph nodes and irregular thickening of the upper lip, the lateral wall of the left nasal vestibule, and the inferior turbinate (Figure 3D,E). Multiple site incisional biopsies were collected from the ulcerated areas: gingiva, palatal, and nasal mucosa. The pathology results described an intense polymorphous lymphoid infiltrate in the epithelium, represented by small- or medium-sized cells, with incised or angulated nuclei and moderate cytoplasm (Figure 4A). Immunohistochemistry (IHC) revealed tumor proliferation with T cells, with the following characteristics: diffuse positive staining for CD3 and CD7, diffuse positive staining for granzyme B, positive staining for CD30, and frequent cells with low-intensity CD56 staining (Figure 4B–E). IHC staining was negative for CD4, CD8, and CD20. The findings were compatible with the diagnosis of extranodal malignant non-Hodgkin lymphoma with T/NK cells.

During the following 3 weeks, until the release of the IHC result, the patient experienced three more episodes of heavy bleeding from the ulcerative oral lesions, requiring emergency surgical control of the bleeding and repeated blood transfusions. The patient was referred to the hematology department for further investigation and treatment. Full-body CT scan and bone marrow biopsy did not detect any additional abnormalities. Thus, disease staging was I-II, and combined chemotherapy with regional radiotherapy was recommended. At the time of this paper’s submission for publication, the patient was initiating chemotherapy.

## 3. Discussions

The COVID-19 outbreak had dramatic demographic, social, and economic consequences worldwide [19]. However, it also revealed some positive aspects of our modern society: the ability of the scientific community to attain rapid and highly efficient solutions when facing an unknown emerging threat to human health, like the novel SARS-CoV-2 virus. Only weeks after the first presumed case of COVID-19 infection, the novel virus was identified as a potential global threat [20]. Furthermore, the most impressive achievement of the scientific community in this difficult context was the rapid development of highly efficient novel vaccines. Thus, after the first year of the COVID-19 pandemic, the most efficient strategy in fighting against infectious maladies—the novel mRNA-based vaccine—was ready for use [21]. Comparative analyses assessing the efficacy of the novel mRNA vaccines (BNT162b2 vaccine and mRNA-1273 vaccine) and viral vector vaccine (Ad26.COV2.S vaccine) revealed superior protection rates for mRNA-based technologies, exceeding 90% [22]. The safety profile related to serious adverse events was similar to classical vaccination methods [23,24]. The most common side effects were local or systemic minor reactions, such as arm pain at the site of injection, fever, and headache [25]. Meanwhile, long-term safety information on mRNA-based vaccines is an ongoing process. The vigilance of clinicians in reporting medical data related to both COVID-19 disease and post-vaccination reactions is fundamental for medical progress and patient safety. The highly potent adaptive immune response achieved through mRNA COVID-19 vaccination is based on complex mechanisms of action, which involve the activation of both B and T cells in secondary lymphoid organs [26]. High-affinity antibody-secreting B cells targeting SARS-CoV-2 viral strains are produced in germinal centers shaped under the direct action of CD4 T cells. After immunization with the BNT162b2 vaccine, increased numbers of specific T cells were found in secondary lymphoid organs and the bloodstream [27]. In addition, the efficacy of protection gained through vaccination is based on germinal center reactions, which are critical for a high-quality B-cell response, and, in mRNA-based vaccines, this type of response was superior to traditional vaccines [28].

In patients diagnosed with hematologic malignancies, an impaired immune response exposes the patient to worse outcomes in the case of SARS-CoV-2 infection, as confirmed by the higher mortality rates reported in this category of patients [29], but also to less efficient protection after vaccination, even when using highly potent agents, such as mRNA vaccines [15,30]. The emergence of hematologic malignancies, such as the non-Hodgkin lymphomas presented in our paper, although only temporarily linked with mRNA COVID-19 vaccination, requires an in-depth investigation into potential underlying interrelations. Until now, isolated cases of newly diagnosed or recurrent hematologic malignancies shortly after COVID-19 vaccination have been reported. Brumfiel et al. presented a case of recurrent cutaneous anaplastic large-cell lymphoma (CD30 positive). The patient developed an ulcerated axillary lesion two days after the first dose of the BNT162b2 vaccine in the ipsilateral arm, raising suspicion of a possible link between these two events [31]. Goldman et al. reported a clinical case of rapid progression in angioimmunoblastic T-cell lymphoma, objectified through serial PET/CT scans performed after the BNT162b2 mRNA vaccine booster dose administration. Several days after the third dose inoculation, the patient noticed a significant enlargement of the cervical lymph nodes. The PET/CT scan revealed increased activity in the pre-existing areas, as well as new hypermetabolic nodal and extranodal sites [17]. Similar isolated cases of lymphoproliferative disorders have been reported after viral vector COVID-19 vaccines as well [32,33].

Lymphadenopathy is included in the list of less common, though possible, side effects associated with mRNA-based vaccines, usually affecting ipsilateral axillary or cervical lymph nodes, corresponding to the site of injection [34]. In most cases, post-vaccination lymphadenopathies had a transient character and did not emerge in the context of malignancy. These have been confirmed as the clinical expression of florid reactive hyperplasia, a marker of the intense immune response which takes place in the germinal centers of the lymph nodes [34,35]. Furthermore, a case of lymphadenopathy suspected as being disease relapse in a lymphoma patient, who achieved complete metabolic response after therapy, turned out to be a false-positive result. After the incisional biopsy, the lymph node enlargement was confirmed as follicular hyperplasia secondary to COVID-19 vaccine administration [36]. To our knowledge, this is the first report of a newly diagnosed large B-cell lymphoma that emerged shortly after administration of the mRNA COVID-19 vaccine. In our opinion, in patients with lymphadenopathy occurring after vaccination, it is important to monitor the spontaneously regressive character of these lesions through regular clinical and imaging assessments. This would allow an early diagnosis of other conditions in lesions exhibiting a progressive character, instead of the typical regressive character specific to reactive lymph nodes.

Oral manifestations have also been reported as uncommon adverse effects after mRNA COVID-19 vaccine inoculation [37,38]. Manfredi et al. reported a case of multiple painful oral ulcers that developed a few days after the first dose of the BNT162b2 vaccine administration [39]. Maeda et al. presented another case of extensive palatal ulcers that emerged 10 days after the second dose of the mRNA-1273 COVID-19 vaccine injection [40]. The lesions from both mentioned cases had a self-limited character, and complete resolution was reported after topical or systemic treatment. No pathological assessment of the oral ulcers was performed in these cases, understandably, due to the progressively healing character of the ulcers. To our knowledge, this is the first report of a severe post-vaccination oral manifestation, immunohistochemically confirmed as T/NK-cell non-Hodgkin lymphoma, emerging within days after mRNA COVID-19 vaccination. Similar to the previous case, there is no causative investigation conducted in our presentation. The link between the two events reported is only temporal. An important aspect to mention is the delay in raising suspicion of a potentially aggressive disease requiring incisional biopsy for the final diagnosis, caused by the atypical extranodal manifestation of the disease. In addition, the aggressiveness of the disease presented in our case, leading to five emergency admissions due to severe hemorrhagic episodes requiring surgical local hemostasis and blood transfusions, emphasizes the importance of rapid diagnosis and treatment.

In conclusion, any clinical event, especially when associated with novel vaccines or treatments, should be reported, as this is the starting point for additional investigations of particular mechanisms of action, thus consolidating knowledge about the safety profile, to the benefit of the patients. 

## Data Availability

The data presented in this study are available on request from the corresponding author.
